# A Climate Change Vulnerability Assessment of California's At-Risk Birds

**DOI:** 10.1371/journal.pone.0029507

**Published:** 2012-03-02

**Authors:** Thomas Gardali, Nathaniel E. Seavy, Ryan T. DiGaudio, Lyann A. Comrack

**Affiliations:** 1 Pacific Coast and Central Valley Group, PRBO Conservation Science, Petaluma, California, United States of America; 2 Emerging Programs and Partnerships Group, PRBO Conservation Science, Petaluma, California, United States of America; 3 Nongame Wildlife Program, California Department of Fish and Game, Sacramento, California, United States of America; Hawaii Pacific University, United States of America

## Abstract

Conservationists must develop new strategies and adapt existing tools to address the consequences of anthropogenic climate change. To support statewide climate change adaptation, we developed a framework for assessing climate change vulnerability of California's at-risk birds and integrating it into the existing California Bird Species of Special Concern list. We defined climate vulnerability as the amount of evidence that climate change will negatively impact a population. We quantified climate vulnerability by scoring sensitivity (intrinsic characteristics of an organism that make it vulnerable) and exposure (the magnitude of climate change expected) for each taxon. Using the combined sensitivity and exposure scores as an index, we ranked 358 avian taxa, and classified 128 as vulnerable to climate change. Birds associated with wetlands had the largest representation on the list relative to other habitat groups. Of the 29 state or federally listed taxa, 21 were also classified as climate vulnerable, further raising their conservation concern. Integrating climate vulnerability and California's Bird Species of Special Concern list resulted in the addition of five taxa and an increase in priority rank for ten. Our process illustrates a simple, immediate action that can be taken to inform climate change adaptation strategies for wildlife.

## Introduction

Climate change poses significant threats to global biodiversity [1]. Our understanding of these threats is in part based on synthetic reviews that summarize the effects that climate change has had on natural systems [2], [3], [4] and on the rapidly growing number of models that provide information on where species, communities, or climatic conditions are predicted to occur under various climate change scenarios [5], [6], [7]. Despite the sophistication of these studies, this information does not, by itself, provide clear direction for conservationists. To address the need for direction, conservation scientists and practitioners have been working at several spatial and temporal scales to find meaningful ways to address the threats of climate change. As a result, there are general management principles and recommendations for adapting management to the projected consequences of climate change [8], [9]. However, such general principles may fail to provide clear on-the-ground guidance in the absence of information about which species and ecosystems are most vulnerable to climate change and how these vulnerabilities interact with non-climate threats and stressors [10].

One approach that appears promising for immediately informing on-the-ground adaptation efforts is to modify existing conservation tools by integrating traditional conservation concerns (such as existing stressors and projected land-use change) with concerns associated with climate change. Traditional conservation planning has relied heavily on lists of at-risk species to guide policy and prioritize conservation actions [9], [11]. These lists typically identify species most in need of conservation action in order to facilitate tailored conservation strategies, optimize and prioritize resource allocation, and build common understanding of impacts and management options [12]. Such lists can be especially effective when they are used to coordinate policy efforts that are broad-scale and span agencies and political boundaries [13].

Integrating climate threats with existing lists of at-risk species requires a framework for defining the relative vulnerability of multiple species to the effects of climate change that will unfold over the next century. Climate change vulnerability assessments (hereafter vulnerability assessments) [10], [11], [14] are designed to assess how susceptible a species or a system is to the negative impacts of climate change in a manner that acknowledges inherent uncertainties in future climatic conditions [14], [15]. Thus, an obvious approach to integrating climate change into existing lists of at-risk species would be to conduct a vulnerability assessment and then incorporate that information into the framework used to rank vulnerability based on more traditional conservation concerns.

Like species worldwide, California's birds are likely to face several climate change-related impacts such as sea level rise and vegetation change [6], [16]. To address the need to include climate vulnerability into bird conservation in California, we developed a vulnerability assessment for California's at-risk birds. In 2008, the California Department of Fish and Game (CDFG) updated its list of at-risk bird species in the California Bird Species of Special Concern monograph (BSSC) [12]. The BSSC identifies 39 species and 24 subspecies or distinct geographic populations for immediate conservation priority. While this list is a valuable tool for many pressing conservation issues, the threat of climate change was not considered when ranking conservation priority. Recognizing this limitation, the authors recommended preparing a supplementary report evaluating the impacts of climate change on current BSSC taxa, those that were nominated for the BSSC but not listed, and those that may be sensitive to climate change but were not nominated for consideration in the original BSSC ranking process.

Our goals were to 1) quantify climate change vulnerability for California's at-risk birds, 2) describe habitat and taxonomic patterns of climate change vulnerability, 3) integrate climate change vulnerability with the existing BSSC list, and 4) provide a simple vulnerability framework and a system for integration with existing at-risk lists for use in other regions.

## Methods

### 2008 Bird Species of Special Concern Process

For the BSSC, experts scored 238 nominated taxa for seven criteria: population size, range size, population trend, range trend, population concentration, percent of range or population in California, and threats. Only realized known threats were considered including habitat loss and degradation, alien species, pollution, overexploitation, and disease. The final BSSC was then compiled by assigning taxa to three levels of priority using both linear and categorical ranking schemes; details of nomination, criteria, and ranking process can be found in the BSSC [12]. Because some bird populations were considered at the species level, whereas others were considered at the subspecies level, we hereafter use the term taxa (or taxon for singular) rather than species to describe the taxonomic units as was done in the BSSC [12].

### Nominations for the Climate Change Vulnerability Assessment

For evaluating climate change vulnerability, we expanded the list of BSSC nominated taxa to include (1) taxa with “high” climate change vulnerability scores as defined by the U.S. national assessment (12 taxa; [17]), (2) taxa projected to suffer a 50% or greater decrease in their California range between 2060 and 2099 under the highest emissions scenario modeled by Audubon California (8 taxa; [18]), or 50% decrease in habitat suitability in California by 2070 under an average of two emission scenarios (4 species; PRBO unpubl. data), (3) taxa that were identified as potentially vulnerable to climate change based on expert opinion (23 taxa), and (4) taxa listed as state and/or federally threatened and endangered or recently delisted (29 taxa). Our final list comprised 358 nominated bird taxa (http://data.prbo.org/apps/bssc/index.php?page=climate-change-vulnerability).

### Climate Change Vulnerability

Our vulnerability assessment for California's birds was designed to inform the state's climate adaptation plans and in particular the revision of the California Wildlife Action Plan. Hence the scale of the analysis is the state of California and the primary users are resource managers and planners. Although there are a growing number of systems available for conducting vulnerability assessments for plants and animals, such as the NatureServe Climate Vulnerability Index [19] and a framework being developed by the U.S. Environmental Protection Agency for Threatened and Endangered Species [20], we opted to develop a new system. We did this in order to take advantage of the wealth of California-specific information on climate change and avian response [6], to tailor and streamline the assessment criteria for birds, and especially to integrate with the existing BSSC list [12].

Vulnerability is generally a measure of the susceptibility or amount of risk of a population to negative impacts [14], [15]. We defined climate vulnerability as the amount of evidence that climate change will negatively impact a population.

To quantify climate vulnerability, we followed existing methodology to consider sensitivity and exposure [14], [21]. Sensitivity is determined by intrinsic traits (such as physiological tolerances) of species that make them vulnerable to climate change. In contrast, exposure is determined by the extrinsic factors (such as increasing temperatures or habitat loss) that will result from climate change. For example, a species that is highly sensitive to increasing temperature would be more vulnerable if the magnitude of climate change (exposure) is larger within that species' geographic range than the same species would be if the magnitude of climate change for its range was smaller [14]. Thus, we scored sensitivity and exposure independently and then multiplied these two scores to generate a climate change vulnerability index.

Sometimes considered a third element of vulnerability, the adaptive capacity of a species to cope with or ameliorate the effects of climate change includes evolutionary changes and plastic ecological responses [14]. We did not include criteria for determining a taxon's adaptive capacity because of the inherent difficulties in scoring adaptive capacity. However, several components of sensitivity can also be considered indirect proxies of adaptive capacity [14], [22], including dispersal ability and habitat specialization, which were captured in our sensitivity component.

### Vulnerability Criteria and Scoring

We developed seven exposure and sensitivity criteria by considering previous work to rank a species' vulnerability to climate change (e.g., NatureServe, Environmental Protection Agency) and information from relevant literature. While one approach would have been to use just one of these existing ranking schemes, we found that because these schemes were generally designed to rank all organisms (not just birds), they were so broad that they incorporated information that was irrelevant for birds (e.g., sex ratio determined by temperature) or failed to fully incorporate more detailed information that is available for birds in California. Hence, we chose to develop a modified scheme that is tailored for California bird populations recognizing that there is not a one-size-fits-all approach to vulnerability assessments [22].

We scored each of the criteria (described below) using information from published papers and the Birds of North America species accounts [23]. Some criteria were more easily scored than others simply because of available information and state of the knowledge of a particular trait. For example, information on migratory status is well-established and hence easily scored. In contrast, information on physiological tolerances is not widely known. When this was the case, our scores were based on the best available information from closely related taxa [14]. After scoring each taxon ourselves, a panel of experts (listed in acknowledgements section) reviewed the scores and suggested changes.

All scores were determined solely with respect to the portion of a taxon's life cycle spent within California; we deemed this approach appropriate for developing a vulnerability assessment system applicable to conservation planning and resource management at the state level and consistent with the 2008 BSSC [12]. Thus, exposure criteria were scored independent of climate change impacts outside of California. For example, sea level rise will likely impact nesting habitat for the Laysan Albatross (*Phoebastria immutabilis*) and other pelagic seabirds, but it probably will not impact their foraging habitat off the coast of California. Similarly, habitat specialization (a sensitivity criterion) was scored according to the habitat requirements of a taxon during the portion of its life cycle spent in California (e.g., species that only occur in California during the winter were scored according to their wintering [non-breeding] habitat requirements).

Below we define each criterion, provide justification for its inclusion, and then follow with details about how each criterion was scored.

### Dealing with Uncertainty

We quantified our uncertainty for each criterion score for each taxon in order to communicate the level of confidence we have for each score. Further, evaluating the collective uncertainty for the criteria should help to identify research needs [10]. Uncertainty arose because of a lack of available information and/or the uncertainty within the quantitative studies [22]. In most cases, our uncertainty scores were based on expert opinion rather than quantitative evidence. The confidence score was assigned qualitatively as:

0 – low confidence
0.5 – moderate confidence
1 – high confidence

### Sensitivity

#### Habitat specialization

Species of birds and butterflies with a high degree of habitat specialization have been shown to be more sensitive to climate change than habitat generalists [24], [25]. Furthermore, habitat specialization has been used as a criterion in efforts to quantify sensitivity to climate change for arctic marine mammals [26], butterflies [27], and in other vulnerability assessment frameworks [22]. Therefore, we scored sensitivity associated with habitat specialization as follows:

1 – low for taxa that use a wide variety of habitat types
2 – moderate for taxa that tolerate some variability in habitat type or element
3 – high for taxa that use only specific habitat types or elements

Habitat specialization scores were determined through a combination of expert opinion and review of each taxon's habitat requirements as described in the Birds of North America species accounts [23]. Habitat specialization was largely defined by nesting requirements for taxa that breed in California, whereas foraging habitat requirements were the determining factors for species that only overwinter in California. For example, two highly specialized taxa are the Western Yellow-billed Cuckoo (*Coccyzus americanus occidentalis*) and Sanderling (*Calidris alba*); the former requires large patches of mature gallery riparian forests for breeding and the latter relies on the wash zone of sandy beaches for winter foraging [28], [29].

#### Physiological tolerances

Some species have very narrow physiological tolerances to climate conditions, such as temperature or water availability. Such physiological limitations may make species less resilient to changing conditions and extreme weather events [30], [31]. We scored sensitivity associated with physiological tolerances as:

1 – low if there was minimal or no evidence of physiological sensitivity to climatic conditions
2 – moderate if there was some evidence of physiological sensitivity to climatic conditions
3 – high if there was strong evidence for physiologically sensitivity to climatic conditions

Published information on the physiological tolerances of North American bird species is very limited. The exceptions include a few studies on owls, which found that certain owl species are particularly sensitive to high temperatures [32], [33]. Additionally, anecdotal evidence suggests that some of California's seabirds, which are not typically exposed to high temperatures, may be sensitive to extreme heat events (PRBO unpubl. data). Beyond these limited accounts, we deduced physiological tolerances using a theoretical approach. Jiguet et al. [31] found that the greater the thermal range normally experienced by a species, the greater its resilience to extreme temperatures. In other words, species adapted to extreme environments (e.g., deserts) are more resilient to extreme temperatures than species adapted to more moderate climates (e.g., coastal areas). Therefore, we scored physiological tolerance based on each taxon's geographic range and its corresponding thermal range.

#### Migratory status

Because migratory birds depend on timing their movements with conditions that facilitate successful survival and reproduction, they are often considered to be more sensitive to changing climatic conditions than species that do not migrate [34], [35], [36]. We scored sensitivity associated with migratory status as:

1 – low for year-round residents
2 – moderate for short-distance migrants (movements primarily restricted to the nearctic zone)
3 – high for long-distance migrants (migrates at least to the neotropics)

The migratory status of all the nominated taxa is widely known and described in literature and field guides, making it relatively straightforward to establish which taxa are resident and which are migratory. However, we established basic migration criteria in order to classify each taxon as either long distance or short distance migrants. The criteria we used were as follows: taxa were classified as long-distance migrants if they migrate between the temperate zone and the tropics (Tropic of Cancer latitude or further south, i.e. southern Mexico, Central and South America); taxa were classified as short-distance migrants if their migrations are restricted to North America/northern Mexico; and taxa were classified as residents if they do not migrate.

#### Dispersal ability

Species with poor dispersal ability, or lack of ability to shift distributions (e.g., geographic barriers, philopatry, neophobia), are less able (or likely) to adapt to spatially shifting conditions, habitats, or resources. Dispersal ability has been included in efforts to quantify sensitivity of climate change for butterflies [27] and it is likely to be important for birds [31]. We scored sensitivity associated with dispersal ability as:

1 – low for taxa with high dispersal ability
2 – moderate for taxa with average dispersal ability
3 – high for taxa with low dispersal ability

Taxa scored with a high dispersal ability include those that are nomadic (irruptive) or migratory and therefore have the capacity to move great distances. Taxa scored with medium dispersal ability were generally non-migratory, though they possess the ability to move moderately long distances between habitat patches. Taxa scored as having low dispersal ability were generally small-sized, non-migratory, sedentary species with a relatively small home range. One useful source of information for evaluating dispersal ability was records of occurrence on the southeast Farallon Island, a 57 ha rocky island located 43 km west of San Francisco where daily bird records have been maintained since 1968 [37]. Birds with the capacity to reach the remote southeast Farallon Island were considered to have high dispersal ability, whereas species that have never been recorded on the island had medium or low dispersal ability if there was no other evidence for long-distance movements.

### Exposure

#### Changes in habitat suitability

Species will be exposed to a wide variety of changes to their habitat including broad scale changes in major vegetation types and changes in key habitat elements. We used existing habitat suitability models (sometimes called probability of occurrence models, species distribution models, or niche models) available for California birds to compare the current habitat suitability in California to the habitat suitability projected for 60–100 years in the future. This criterion includes the effects of sea-level rise. We scored exposure associated with habitat as:

1 – low if habitat suitability is expected to increase or decrease by 0–10%
2 – moderate if habitat suitability is expected to decrease by 10–50%
3 – high if habitat suitability is expected to decrease by >50%

For scoring change in habitat suitability, we primarily relied on habitat suitability models independently developed by Audubon California [18] and PRBO Conservation Science [6]. The Audubon California and PRBO Conservation Science models both used emissions trajectories taken from the Intergovernmental Panel on Climate Change SRES A2 scenario [38]. If the Audubon and PRBO models agreed with each other in magnitude and direction, and were consistent with expert opinion, the resulting habitat score was given a high level of confidence. Conversely, if the models contradicted each other, the score was decided by expert opinion and given a low to medium level of confidence. Expert opinion was also used to interpret model results or for where models were not available. Expert opinion was informed by an extensive literature review of projected climate change effects in California [39]. Habitat suitability models were not available for most waterbirds, however climate models suggest that water availability will decrease and thus freshwater wetland habitat will decline throughout California [39], [40]. Freshwater wetland dependent taxa were therefore all given a habitat change score of 2 (10–50% change in habitat). Taxa largely restricted to tidal marsh, tidal mudflats, coastal beach strand, and rocky intertidal zones will likely be impacted by sea level rise and were therefore assigned a habitat change score of 3 (>50% change in habitat).

#### Changes in food availability

Species may be exposed to climate change effects if the timing, availability, and abundance of critical food resources are altered. The linkage between climate change effects of food availability and changes in reproductive success or survival is believed to be important for some birds such as seabirds [41]. We scored exposure to changes in food availability as:

1 – low if food availability for a taxon would be unchanged or increase
2 – moderate there was evidence that food availability may decrease
3 – high if there was evidence that there would be major decreases in food availability

According to current projections, seabirds depending on the seasonally productive California Current may experience significant reductions in food supply due to increasing ocean acidification and delayed upwelling during the breeding season [39], [41]. Therefore, we scored food supply declines for most seabirds as moderate to high, depending on the diet specialization of each taxon. The confidence for these scores, however, is low given the uncertainty of these oceanographic predictions. For most terrestrial species, virtually no information exists on how climate change will affect food supply. The food and habitat of certain species are inextricably linked; for example, sagebrush is both the primary habitat component and food source for Greater Sage-Grouse (*Centrocercus urophasianus*) [42]. However, the relationship between climate and food for most landbird species is often more complicated. For insectivorous birds, climate change may actually increase invertebrate prey populations because invertebrate productivity generally increases with an increase in temperature [43], though the long-term effects of climate change on invertebrate prey populations is unknown.

#### Changes in extreme weather

Survival and fecundity of some species are impacted by extreme weather events [44], [45]. We scored exposure to extreme weather as:

1 – low if there is no evidence that a taxon would be exposed to more frequent or severe extreme weather events
2 – moderate if a taxon is expected to be exposed to some increase in extreme weather events
3 – high if a taxon is very likely to be exposed to major increases in the number and duration of extreme weather events

Extreme weather could mean exceptionally stormy weather, storms outside normal seasons, and/or prolonged conditions such as drought and unusually high temperatures. We relied on the climate change literature for California to inform our assessment of the extreme weather each taxon may experience throughout its geographic range [39]. Of all the ecoregions in California, the deserts, Central Valley, and low elevation Sierra Nevada are predicted to experience the most extreme hot weather events for extended periods, therefore the taxa of those ecoregions were assigned high extreme weather scores. Coastal and tidal marsh taxa scored moderate to high due to the potential for increased exposure to coastal storms and tidal flooding.

### Climate Vulnerability, Ranking, and Integration

We began by quantifying climate change vulnerability for the full nominated set of taxa. For each taxon, we multiplied the sum of the exposure scores by the sum of the sensitivity scores to generate a climate vulnerability index (higher index indicated greater vulnerability). In order to cull the full set of nominated taxa into those most vulnerable to climate change, we identified those taxa with the highest 25% of all scores as vulnerable to climate change. We further ranked these species into three levels of climate change priority (high, moderate, and low) by identifying natural breaks in the distribution of vulnerability scores.

To integrate the two lists, BSSC and climate change prioritized, we took a similar approach to that proposed by the U.S. Environmental Protection Agency to integrate climate change vulnerability with existing stressors for threatened and endangered species [20]. We developed a matrix that combines the priority ranks from each list to produce a final integrated list (Table 1). A priority 1 taxon on the BSSC remains priority 1 on the final integrated list regardless of its climate change priority, even if it was not prioritized by the vulnerability assessment. A BSSC priority 2 taxon was raised to priority 1 on the integrated list if it was ranked as priority 1 in the vulnerability assessment because the effects of climate change are likely to exacerbate the factors that make it at-risk. Similarly, a BSSC priority 3 was raised to priority 2 on the integrated list if it was scored as priority 1 or 2 in the vulnerability assessment. Taxa that were not prioritized by the BSSC could be added to the integrated list in two ways: (1) species that were priority 1 in the vulnerability assessment were added to the integrated list regardless of their original BSSC score, (2) taxa with an original BSSC score that was close to prioritization (original score ≥30) were added to the list if their vulnerability was priority 2.

**Table 1:** Matrix that integrates the California Bird Species of Special Concern ranks with the climate change vulnerability assessment ranks to generate three levels of priority that represent new Bird Species of Special Concern ranks that include the threat of climate change.

	Climate Change Vulnerability Priority Rank		
BSSC Priority Rank	1	2	3
1	1	1	1
2	1	2	2
3	2	2	3
Unranked	3	Unranked	Unranked
Unranked with score >30	3	3	Unranked

## Results

### Sensitivity, Exposure, and Confidence

**Figure 1:**
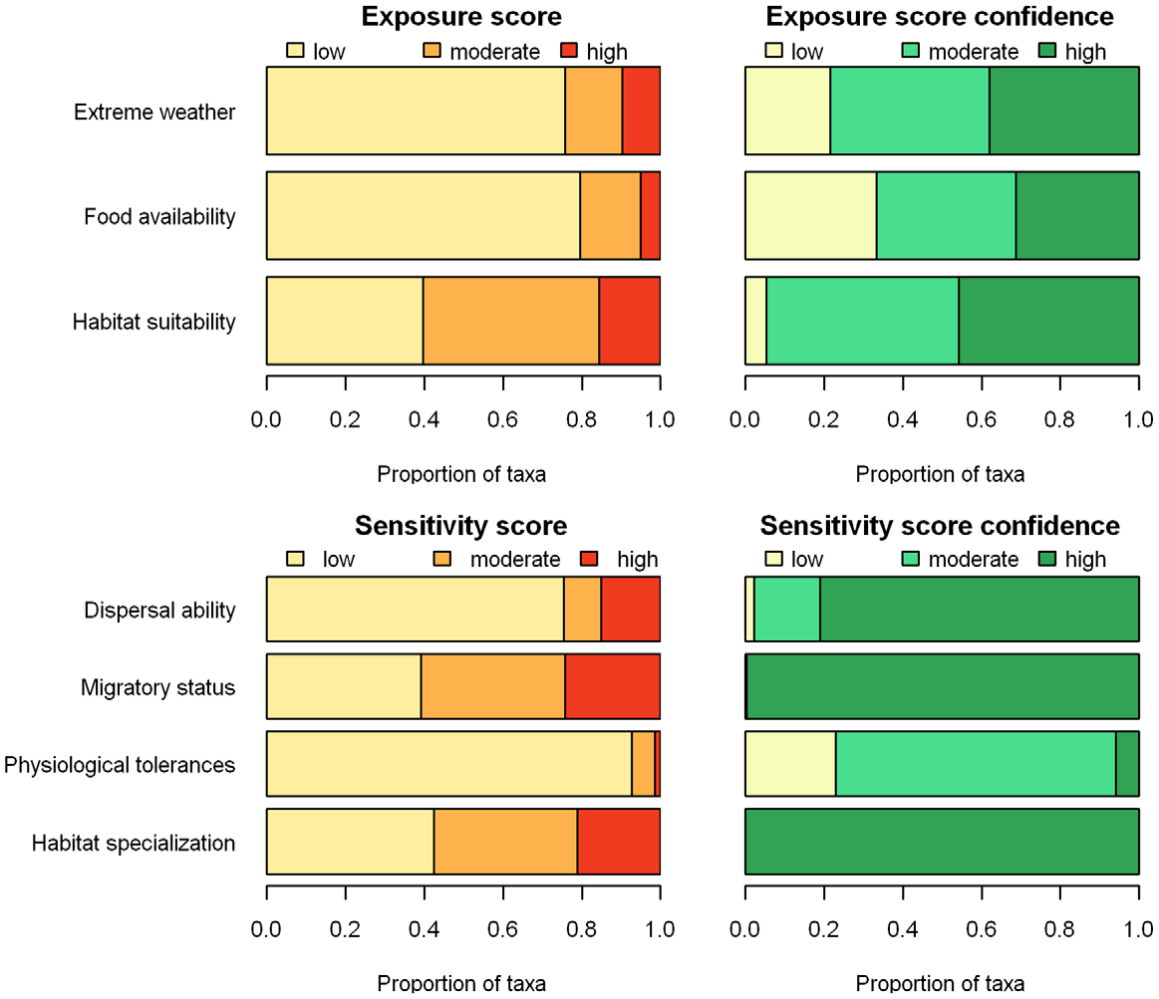
Sensitivity and exposure criteria scores and confidence scores for 358 bird taxa from California.

We scored the exposure and sensitivity criteria for a total of 358 bird taxa in California (http://data.prbo.org/apps/bssc/index.php?page=climate-change-vulnerability). For the three exposure criteria, scores were highest for habitat suitability, with 61% of taxa scoring moderate or high, indicating that habitat suitability is likely to decrease for the majority of taxa (Fig. 1). For food availability and extreme weather, we scored only 20–25% of taxa as moderate or high (Fig. 1). We had the greatest confidence in our scores for changes in habitat suitability followed by extreme weather and then food availability scores (Fig. 1).

For the four sensitivity criteria, scores were highest for the migratory status and habitat specialization categories, with approximately 60% of taxa scored as moderate or high for these criteria. Sensitivity associated with dispersal ability was scored as moderate or high for 25% of the taxa. For physiological tolerances, only 7% of the taxa were scored as moderate or high (Fig. 1). Confidence for sensitivity scores was generally high, with the exception of physiological tolerances, for which we scored 94% as low or moderate (Fig. 1).

### Climate Change Vulnerability List

**Figure 2:**
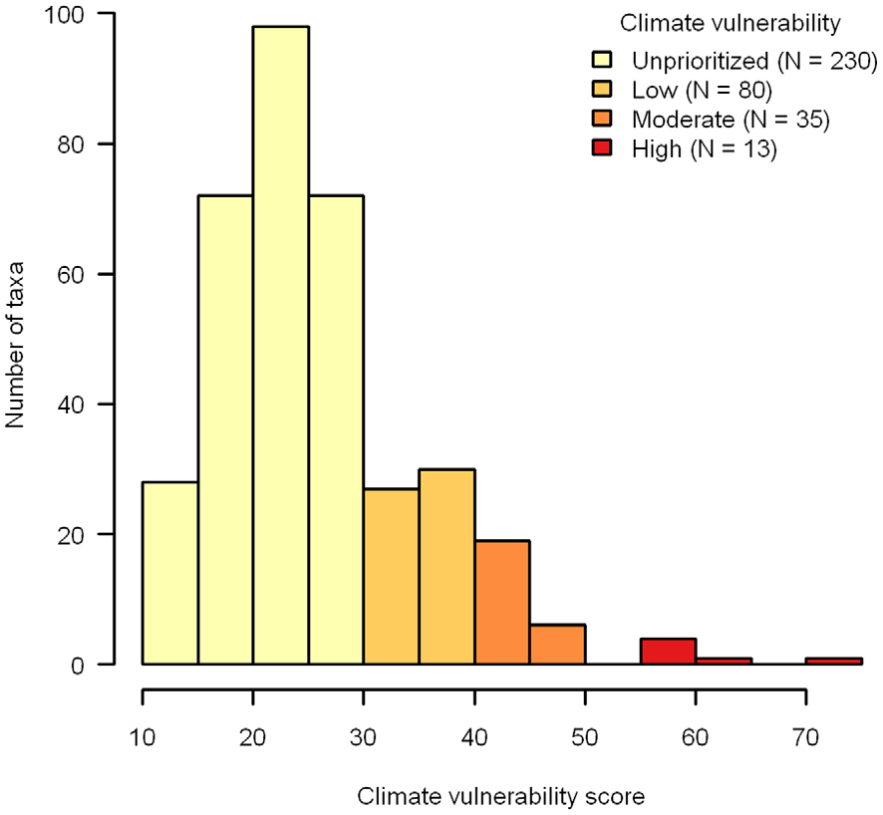
Total climate change vulnerability scores for 358 bird taxa in California; those taxa with scores <30 are currently unprioritized, ≥30 and <40 are low priority, ≥40 and <45 are of moderate priority, and ≥40 are high priority.

Climate change vulnerability scores for the 358 nominated taxa ranged from 12 to 72, and had a right-skewed distribution with a median score of 24 (Fig. 2). The 3^rd^ quartile (75% of all observations) fell at a vulnerability score of 30. However, because this score was shared by multiple taxa, including all taxa with a score of 30 or greater accounted for 128 (35%) of the nominated taxa. Within the group of 128 prioritized taxa, we ranked 80 taxa as low priority (priority 3; scores 30–39), 35 taxa as moderate priority (priority 2; scores 40–44), and 13 taxa as high priority (priority 1; scores 45–72; Fig. 2).

**Figure 3:**
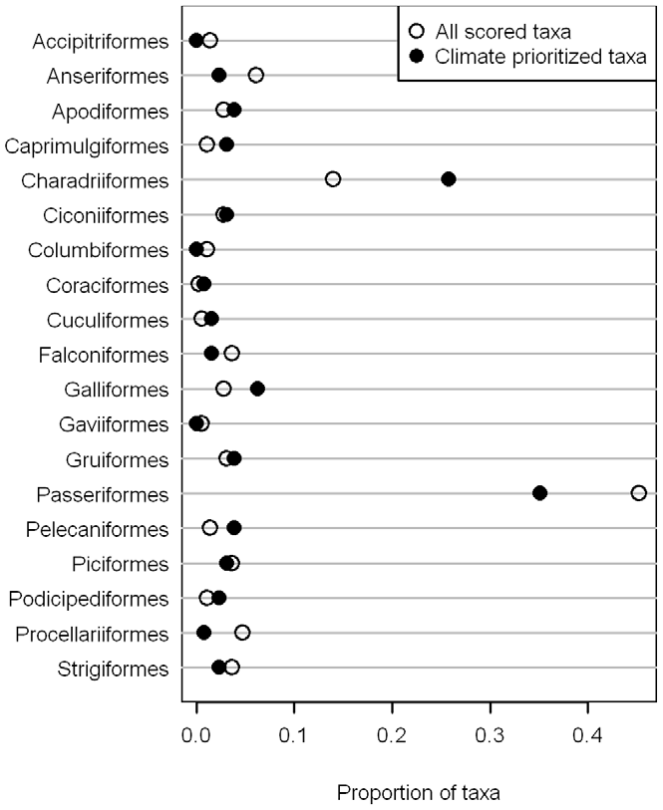
Proportion of taxa in each avian order that were on the full nominated list (hollow circles) compared with the proportion (of those on the full list) that were classified as climate vulnerable (solid circles). Orders for which the distance between the two circles is larger are ones that had a higher proportional vulnerability from climate change.

The representation of avian orders varied dramatically in both the list of all taxa that were scored (nominated taxa list) and the list of prioritized taxa (Fig. 3). Comparing the proportion of taxa between the list of all taxa scored and the prioritized list provides information about the degree to which an order was prioritized disproportionately relative to its abundance on the original list. For example, Passeriformes comprised 45% of the nominated taxa list, yet only represented 35% of the prioritized list. Conversely, Charadriiformes made up a greater proportion of the prioritized list (26%) compared to their representation in the list of all nominated taxa (14%). Though the difference was less extreme, Galliformes, Caprimulgiformes, and Pelecaniformes also comprised a greater proportion of the prioritized taxa than of the nominated taxa list (Fig. 3).

**Figure 4:**
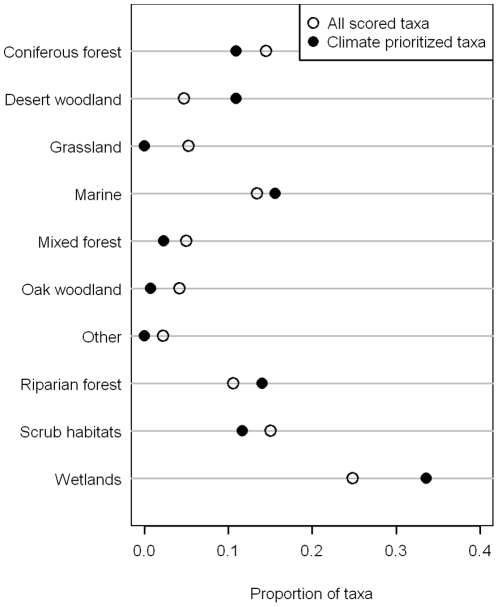
Proportion of taxa in habitat groupings that were on the full nominated list (hollow circles) compared with the proportion (of those on the full list) that were classified as climate vulnerable (solid circles). Groups for which the distance between the two circles is larger are ones that had a higher proportional vulnerability from climate change. Habitat classification follows Shuford and Gardali (2008): Marine (nearshore, offshore, and pelagic waters), Wetlands (tidal flats, tidal marsh, freshwater marsh, wet meadows, vernal pools, flooded agricultural fields, and riverine, lacustrine, and estuarine waters), Riparian forest and woodland, Coniferous forest, Mixed Forest (evergreen hardwood forest), Oak woodland and oak savanna, Desert woodland (Joshua tree, fan palm, Mohave yucca, ocotillo, and pinyon-juniper), Scrub habitats (chaparral, coastal scrub, desert scrub, and sagebrush scrub), and Grassland (native grassland, pastureland, grass-like crops, weedy fields, and sparsely-vegetated cultivated fields).

The representation of habitat affinities also varied across both lists (Fig. 4). Most notably, wetland taxa comprised 25% of the nominated taxa list, but 34% of the prioritized list. Other habitat affinities that comprised a greater proportion of the prioritized list were species associated with desert woodlands, marine areas, and riparian forests (Fig. 4).

### Integrated BSSC List

Ten taxa were raised in priority on the integrated list; three taxa went from priority 2 to priority 1 (Greater Sage Grouse, Yellow Rail [winter], Alameda Song Sparrow) and seven taxa went from priority 3 to priority 2 (Suisun Song Sparrow, Samuel's Song Sparrow, Snowy Plover [interior population], Cassin's Auklet, Bendire's Thrasher, San Francisco Common Yellowthroat, Modesto Song Sparrow). Five taxa were new to the list and ranked as priority 3 (Black Oystercatcher, Scott's Oriole, Royal Tern, Elegant Tern, and Rhinoceros Auklet). Scientific names are listed in Table 2.

**Table 2:** List of species, subspecies, and distinct populations that were classified as vulnerable to the impacts of climate change in California.

Common name	Scientific name	Climate vulnerability score	Climate Priority	Status^a^
Greater Sage-Grouse	*Centrocercus urophasianus*	72	1	2
Yellow Rail (winter)	*Coturnicops noveboracensis*	49	1	2
California Black Rail	*Laterallus jamaicensis coturniculus*	49	1	ST
California Clapper Rail	*Rallus longirostris obsoletus*	56	1	SE, FE
Yuma Clapper Rail	*Rallus longirostris yumanensis*	48	1	ST, FE
Black Oystercatcher	*Haematopus bachmani*	48	1	
California Least Tern	*Sternula antillarum browni*	63	1	SE, FE
Marbled Murrelet	*Brachyramphus marmoratus*	48	1	SE, FT
Elf Owl	*Micrathene whitneyi*	45	1	SE
Suisun Song Sparrow	*Melospiza melodia maxillaries*	56	1	3
Samuel's Song Sparrow	*Melospiza melodia samuelis*	56	1	3
Alameda Song Sparrow	*Melospiza melodia pusillula*	56	1	2
Scott's Oriole	*Icterus parisorum*	48	1	
American White Pelican	*Pelecanus erythrorhynchos*	40	2	1
California Brown Pelican	*Pelecanus occidentalis californicus*	42	2	
Brandt's Cormorant	*Phalacrocorax penicillatus*	42	2	
Pelagic Cormorant	*Phalacrocorax pelagicus*	42	2	
Swainson's Hawk	*Buteo swainsoni*	42	2	ST
Light-footed Clapper Rail	*Rallus longirostris levipes*	40	2	SE, FE
Snowy Plover (interior population)	*Charadrius nivosus*	42	2	3
Snowy Plover (coastal population)	*Charadrius nivosus*	42	2	FT
Wandering Tattler	*Tringa incana*	42	2	
Ruddy Turnstone	*Arenaria interpres*	42	2	
Black Turnstone	*Arenaria melanocephala*	42	2	
Surfbird	*Aphriza virgata*	42	2	
Red Knot	*Calidris canutus*	40	2	
Sanderling	*Calidris alba*	40	2	
Black Tern	*Chlidonias niger*	40	2	2
Royal Tern	*Thalasseus maximus*	42	2	
Elegant Tern	*Thalasseus elegans*	42	2	
Common Murre	*Uria aalge*	40	2	
Pigeon Guillemot	*Cepphus Columba*	40	2	
Xantus's Murrelet	*Synthliboramphus hypoleucus*	40	2	ST
Craveri's Murrelet	*Synthliboramphus craveri*	40	2	
Cassin's Auklet	*Ptychoramphus aleuticus*	40	2	3
Rhinoceros Auklet	*Cerorhinca monocerata*	40	2	
Tufted Puffin	*Fratercula cirrhata*	40	2	1
Western Yellow-billed Cuckoo	*Coccyzus americanus occidentalis*	40	2	SE
Great Gray Owl	*Strix nebulosa*	40	2	SE
Brown-crested Flycatcher	*Myiarchus tyrannulus*	42	2	
Arizona Bell's Vireo	*Vireo bellii arizonae*	40	2	SE
Least Bell's Vireo	*Vireo bellii pusillus*	40	2	SE, FE
Bendire's Thrasher	*Toxostoma bendirei*	42	2	3
San Joaquin Le Conte's Thrasher	*Toxostoma lecontei macmillanorum*	40	2	1
San Francisco Common Yellowthroat	*Geothlypis trichas sinuosa*	42	2	3
Inyo California Towhee	*Melozone crissalis eremophilus*	42	2	SE, FT
Modesto Song Sparrow	*Melospiza melodia mailliardi*	42	2	3
Gray-crowned Rosy Finch	*Leucosticte tephrocotis*	42	2	
Fulvous Whistling-duck	*Dendrocygna bicolor*	30	3	1
Bufflehead	*Bucephala albeola*	35	3	
Barrow's Goldeneye	*Bucephala islandica*	35	3	
Mountain Quail	*Oreortyx pictus*	30	3	
Little San Bernadino Mountain Quail	*Oreortyx pictus russelli*	30	3	
Inyo California Quail	*Callipepla californica canfieldae*	30	3	
Gambel's Quail	*Callipepla gambelii*	36	3	
Ruffed Grouse	*Bonasa umbellus*	35	3	
Mount Pinos Sooty Grouse	*Dendragapus fuliginosus howardi*	35	3	2
Sooty Grouse	*Dendragapus fuliginosus*	30	3	
Eared Grebe	*Podiceps nigricollis*	30	3	
Western Grebe	*Aechmophorus occidentalis*	30	3	
Clark's Grebe	*Aechmophorus clarkia*	30	3	
Black Storm-petrel	*Oceanodroma melania*	32	3	3
Double-crested Cormorant	*Phalacrocorax auritus*	36	3	
American Bittern	*Botaurus lentiginosus*	30	3	
Least Bittern	*Ixobrychus exilis*	35	3	2
White-faced Ibis	*Plegadis chihi*	35	3	
Wood Stork	*Mycteria Americana*	30	3	1
Osprey	*Pandion haliaetus*	35	3	
Willet (winter)	*Tringa semipalma*	30	3	
Whimbrel	*Numenius phaeopus*	35	3	
Alaska Marbled Godwit	*Limosa fedoa beringia*	30	3	
Short-billed Dowitcher	*Limnodromus griseus*	35	3	
Wilson's Phalarope	*Phalaropus tricolor*	35	3	
Red-necked Phalarope	*Phalaropus lobatus*	36	3	
Red Phalarope	*Phalaropus fulicarius*	30	3	
Heermann's Gull	*Larus heermanni*	30	3	
Gull-billed Tern	*Gelochelidon nilotica*	36	3	3
Caspian Tern	*Hydroprogne caspia*	30	3	
Forster's Tern	*Sterna forsteri*	30	3	
Black Skimmer	*Rynchops niger*	30	3	3
Greater Roadrunner	*Geococcyx californianus*	35	3	
Northern Saw-whet Owl	*Aegolius acadicus*	32	3	
Lesser Nighthawk	*Chordeiles acutipennis*	30	3	
Common Nighthawk	*Chordeiles minor*	30	3	
Common Poorwill	*Phalaenoptilus nuttallii*	30	3	
Mexican Whip-poor-will	*Caprimulgus arizonae*	36	3	
Black Swift	*Cypseloides niger*	32	3	3
Vaux's Swift	*Chaetura vauxi*	35	3	2
Costa's Hummingbird	*Calypte costae*	36	3	
Broad-tailed Hummingbird	*Selasphorus platycercus*	30	3	
Rufous Hummingbird	*Selasphorus rufus*	30	3	
Belted Kingfisher	*Megaceryle alcyon*	36	3	
Gila Woodpecker	*Melanerpes uropygialis*	30	3	SE
Black-backed Woodpecker	*Picoides arcticus*	35	3	
Gilded Flicker	*Colaptes chrysoides*	30	3	SE
Pileated Woodpecker	*Dryocopus pileatus*	30	3	
Southwestern Willow Flycatcher	*Empidonax traillii extimus*	32	3	SE, FE
Vermilion Flycatcher	*Pyrocephalus rubinus*	30	3	2
Gray Vireo	*Vireo vicinior*	36	3	2
Gray Jay	*Perisoreus canadensis*	30	3	
Yellow-billed Magpie	*Pica nuttalli*	30	3	
Bank Swallow	*Riparia riparia*	32	3	ST
Black-capped Chickadee	*Poecile atricapillus*	30	3	
Juniper Titmouse	*Baeolophus ridgwayi*	30	3	
Verdin	*Auriparus flaviceps*	36	3	
Cactus Wren	*Campylorhynchus brunneicapillus*	36	3	
San Diego Cactus Wren	*Campylorhynchus b. sandiegensis*	35	3	1
Clark's Marsh Wren	*Cistothorus palustris clarkae*	36	3	2
Coastal California Gnatcatcher	*Polioptila californica californica*	32	3	FT
Black-tailed Gnatcatcher	*Polioptila melanura*	36	3	
Swainson's Thrush	*Catharus ustulatus*	30	3	
California Swainson's Thrush	*Catharus ustulatus oedicus*	35	3	
Varied Thrush	*Ixoreus naevius*	35	3	
Crissal Thrasher	*Toxostoma crissale*	30	3	3
Le Conte's Thrasher	*Toxostoma lecontei*	35	3	
Virginia's Warbler	*Oreothlypis virginae*	30	3	
Lucy's Warbler	*Oreothlypis luciae*	30	3	3
Sonora Yellow Warbler	*Setophaga petechia sonorana*	35	3	2
Abert's Towhee	*Melozone aberti*	35	3	
Brewer's Sparrow	*Spizella breweri*	30	3	
Belding's Savannah Sparrow	*Passerculus sandwichensis beldingi*	30	3	SE
Large-billed Savannah Sparrow	*Passerculus sandwichensis rostratus*	35	3	2
Fox Sparrow	*Passerella iliaca*	30	3	
Stephens's Fox Sparrow	*Passerella iliaca stephensi*	30	3	
Lincoln's Sparrow	*Melospiza lincolnii*	35	3	
Hepatic Tanager	*Piranga flava*	36	3	
Pine Grosbeak	*Pinicola enucleator*	30	3	
Red Crossbill	*Loxia curvirostra*	30	3	

a Status refers taxa listed as threatened or endangered by state or federal law. st = state threatened, se = state endangered, ft = federally threatened, fe = federally endangered. Numbered designations indicate California Bird Species of Special Concern priority levels within the list (1, 2, or 3; highest to lowest).

### BSSC, State, and Federally Threatened and Endangered Species

A total of 29 bird taxa are listed as federally and/or state threatened and endangered in California. Of the 29 listed taxa, fully 21 (72%) are considered vulnerable to climate change in California (Table 2). The eight threatened and endangered species that were not on the climate change vulnerability list include Short-tailed Albatross (*Phoebastria albatrus*), San Clemente Loggerhead Shrike (*Lanius ludovicianus mearnsi*), Northern Spotted Owl (*Strix occidentalis caurina*), San Clemente Sage Sparrow (*Amphispiza belli clementeae*), Willow Flycatcher (*Empidonax traillii*), Bald Eagle (*Haliaeetus leucocephalus*), California Condor (*Gymnogyps californianus*), and Greater Sandhill Crane (*Grus canadensis tabida*).

Thirty one of the 63 (49%) taxa on the 2008 BSSC list were also on the prioritized climate change vulnerability list (Table 2).

## Discussion

There are many types of studies that assess the vulnerability of a species or system to climate change. For example, studies that model the potential impacts of sea level rise on bird habitat can be considered a vulnerability assessment [16]. Our vulnerability assessment however uses a system to score multiple taxa and results in a ranked index of vulnerability. Our results represent the first effort to quantify the climate vulnerability of California's birds and are to our knowledge one of the first efforts to integrate climate vulnerability into an existing list of at-risk species [20]. Given the novelty of climate vulnerability, it is important to consider how our approach compares to other efforts to quantify climate vulnerability, whether there are certain groups of birds that are more vulnerable than others, and the ways in which our process may be improved or applied to other situations.

### Comparison with Other Vulnerability Assessments

We know of no similar peer-reviewed climate change vulnerability assessments for birds. The one report that does exist was done at a different geographic scale – the United States of America (US) [17]. The US assessment provided results by broad habitat groupings or geography which makes comparisons to our assessment difficult. Although we also summarized our results by habitat, the group classifications and their constituent species differed considerably between the two systems. Comparison to the US assessment is further complicated because their results were only provided for a subset of full species (available online only) while our assessment considered species, sub-species, and distinct populations consistent with the BSSC [12] and the US Endangered Species Act. Nevertheless, some general comparisons can be made. The highest percentages of vulnerable species in the US assessment were found in “ocean” and “coastal” environments. Marine-associated taxa were the second most vulnerable group in our assessment. We did not have an equivalent coastal category but our results clearly show taxa that use coastal environments are vulnerable to climate change in California. These taxa are those that use rocky shorelines, beaches, coastal wetlands and estuaries, and nearshore waters. Species associated with wetlands in the US assessment were not particularly vulnerable yet they were by far the most vulnerable group in our analysis. We hypothesize that the types of wetlands considered, the constituent taxa, and the different scales of analysis may explain the differences between these vulnerability analyses.

The methodological approach between the two systems was similar in that both had criteria for dispersal, migratory status, and habitat specialization (the US had two criteria that ours did not– breeding habitat obligate and niche specificity). The US system had only one relatively generic exposure criterion that differed considerably from our approach, which considered three. Another noteworthy difference was that the US assessment had a sensitivity score for reproductive potential and we did not. Finally, the US assessment scored all criteria using expert opinion while we used a combination of quantitative information (e.g., habitat suitability models), literature, and expert opinion. Given these differences, it is encouraging that some of the general patterns were similar; this suggests that vulnerability assessments developed at dramatically different scales may provide information that can guide conservation at other scales.

### Habitat and Taxonomic Patterns

The disproportionate representation of vulnerable taxa associated with wetlands is not surprising given this habitat will likely be heavily impacted by sea level rise and extreme climatic events, and because water availability is predicted to decrease thus reducing freshwater wetlands throughout the state [39], [40]. California also contains several taxa that are restricted almost completely to wetlands and show a high degree of specialization within this broad habitat category. For example, three sub-species of Song Sparrow are endemic to the tidal-marshes of the San Francisco Bay. Climate change impacts to wetlands and wetland birds go beyond habitat loss; wetlands may be impacted by alteration of timing of recharge, extreme climatic events, and changes in plant communities and prey base [45].

Relative to wetlands, no other habitat group stood out as especially vulnerable to climate change. The marine group contained the second highest proportion of vulnerable taxa followed closely by riparian. On the other end of the spectrum, grassland and oak woodland taxa were the least vulnerable to climate change. The low representation of vulnerable taxa within grasslands is not surprising given the prediction that this habitat type will likely increase in the state [39], [46]. Oak woodlands may increase as well but the predictions are not as consistent as they are for grasslands [39], [46].

Two taxonomic groups had by far the highest proportion of vulnerable taxa: Charadriiformes and Passeriformes. Charadriiformes were considered vulnerable in a far higher proportion relative to the total number scored. It is not surprising that Charadriiformes show high numbers of vulnerable taxa in California given that this order contains shorebirds that rely on wetlands, rocky shorelines, and beaches as well as seabirds that nest on rocky shorelines, and are also sensitive to changes in the pelagic food web. More Passeriformes were scored than any other order, thus the high number of taxa that were ranked as vulnerable was not disproportionate to the number of taxa that was scored.

### Listed Species

Our analysis indicates that the majority (72%) of threatened and endangered species in California are vulnerable to climate change. Climate change is likely to exacerbate current stressors, further increasing extinction probability for these already imperiled taxa [47], [48]. In California, many listed species are wetland-specialists, which is not surprising given this habitat has one of the highest rates of loss in California. Estimates indicate that California has lost over 90% of its original wetlands [49] and what remains is highly fragmented. Given that our assessment indicates that wetland associated taxa are highly vulnerable to climate change, it is not surprising that so many threatened and endangered taxa are also vulnerable.

Some threatened and endangered taxa not identified as vulnerable by our assessment may actually be vulnerable at different scales such as those that cover their entire distribution. For example, we did not consider Short-tailed Albatross as vulnerable to climate change in California, where it occurs exclusively and rarely as a non-breeding visitor at sea, but it may be vulnerable on its breeding grounds due to sea level rise and other factors.

That only half of the 2008 BSSC species were also considered vulnerable to the impacts of climate change suggests that conservation measures could be successful at arresting existing population declines and range retractions. On the other hand, priority should be given to those taxa on both lists in order to formulate short- and long-term conservation measures.

### Limits of Our Approach

Our approach to assessing climate change vulnerability benefited from extensive review of comparable systems [19], [20], an overview of vulnerability assessments [22], and published literature on vulnerability [14]. Still, our approach has several limitations. The scale of our assessment is California, which is suitable for the target audience, but may not identify taxa vulnerable at scales larger or smaller. Further, we only scored taxa during their primary role in California as was done in the BSSC [12], which may not identify taxa vulnerable during a life stage occurring outside of the state. Because we followed the process developed by the Technical Advisory Committee for the 2008 BSSC [12], not all bird taxa in California were considered and scored. Hence, it is possible that some taxa not considered in our assessment are vulnerable to climate change but not identified. However, we used several means to expand the list of nominated taxa (see methods) and believe our assessment evaluates California's vulnerable taxa sufficiently.

Our system did not take into account adaptive capacity, a component that determines a taxon's or a system's vulnerability to climate change. Adaptive capacity is used two ways in the vulnerability assessment literature: primarily to describe the capacity (evolutionarily or plasticity) of an organism to accommodate climate change impacts with minimal disruption, but also to describe conservation strategies that are designed to aid a species or a system to prepare for and cope with the impacts of climate change [14]. We judged adaptive capacity, in either sense, to be too difficult to score given how little information and guidance exists upon which to make objective assessments. Moreover, several components of sensitivity can also be considered indirect proxies of adaptive capacity [14], [22] including dispersal ability and habitat specialization, which were captured in our sensitivity component. At this time, we do not believe that excluding adaptive capacity limits the utility of our climate vulnerability assessment. However, as more information on adaptive capacity becomes available, it may warrant inclusion in future assessments.

We did not include any component of reproductive strategy as a sensitivity criterion as has been done in other systems [16], [21]. Some studies suggest that low fecundity coupled with long generation times makes a taxon more susceptible to extinction under climate change [50]. However, long-lived taxa have more opportunities to reproduce and hence may be able to recover from bad years. It has also been suggested that birds with higher fecundity might not live long enough to be able to adapt to directional changes in their environment [51]. Furthermore, when avian body size has been used as a surrogate for reproductive strategy, it has not been found to be a predictor of vulnerability to global change [25]. Hence, it was not clear to us how climate change will impact taxa with different reproductive strategies and we judged it best to exclude it. However, as more information on reproductive strategy becomes available, it may warrant inclusion in future assessments.

We quantified uncertainty only as a means to communicate about the relative confidence we had in each score and to identify research needs. Some ranking systems use uncertainty scores to weight a particular criterion with more or less emphasis depending on the goals of the system. The merits of including uncertainty formally into estimates of climate vulnerability should be considered in the future.

We used a linear ranking scheme and set an arbitrary cut-off to classify taxa into three levels of priority. We considered the relative merits and short-comings of various ranking schemes [52], [53] and judged a simple linear approach to be sufficient. In particular, we value the ease at which it can be applied and understood by a wide range of users. We acknowledge that our numerical cutoff point, between being prioritized as vulnerable or not, is arbitrary but believe that any ranking system suffers from this short-coming when the goal is to develop a prioritized list. We recommend users consult the full set of scores and we have made them available for download (http://data.prbo.org/apps/bssc/index.php?page=climate-change-vulnerability). We further encourage users to develop alternate approaches to ranking and make comparisons to our system.

### Fostering Conservation

Lists of at-risk taxa have long been tools for conservation and their efficacy has been debated since their inception [54]. Unlike traditional at-risk lists, climate change vulnerability assessments like ours attempt to look well to the future and hence can facilitate conservation decisions at time scales much longer than traditionally considered [22]. Unlike traditional lists, climate change vulnerability assessments like ours do not exclusively identify taxa with the highest extinction probabilities (i.e., a taxon can be vulnerable but not necessarily at risk of extinction). Hence, because species with high immediate extinction probabilities may be poor conservation investments [54], vulnerability assessments that result in ranked lists of vulnerable taxa can provide a more efficient and effective way to spend limited resources.

Like traditional lists, climate change vulnerability assessments by themselves do little for conservation; they must be part of conservation planning efforts. Conservation planning efforts typically consist of elements that identify conservation targets, assess risks and vulnerability of those targets, identify management options, implement management options, and, ideally, continuously monitor, review, and revise the process [22]. Integrating climate vulnerability into the BSSC list takes the first step in this process. We purposely did not allow any BSSC-listed taxa to be lowered in priority or removed from the list; given uncertainty inherent in all ranking systems, we judged it better to recommend more conservation priority rather than too little. The integration resulted in adding five taxa and raising priority for ten.

Our vulnerability assessment of California's birds and its integration with the CDFG's Bird Species of Special Concern list [12] was undertaken to inform the revision of the California Wildlife Action Plan originally completed in 2007 and updated every 5 to 10 years [55]. Although the original California Wildlife Action Plan identified climate change as a concern, it did not go into sufficient detail about how climate change could impact the state's wildlife or identify associated management actions. CDFG is in the beginning stages of revising the plan to better address climate change impacts and ways to address them. In addition to the California Wildlife Action Plan, the vulnerability assessment and integrated list can benefit other conservation planning efforts (e.g., Joint Ventures) by helping to prioritize species and habitats where conservation actions are most needed.

Climate change does not act alone in threatening biodiversity. Biodiversity is threatened by familiar stressors such as habitat loss and degradation, invasive species, pollution, over-exploitation, and disease. Climate change exacerbates these familiar stressors which, together, are predicted to cause mass global extinctions [1]. To focus only on the threat of climate change steals the focus from lasting conservation actions that must consider all threats simultaneously. A climate change vulnerability assessment is a useful tool but only if brought to the repair with a complete toolbox.
